# The Skin in Cowden Syndrome

**DOI:** 10.3389/fmed.2021.658842

**Published:** 2021-06-10

**Authors:** Agnes Lim, Joanne Ngeow

**Affiliations:** ^1^Cancer Genetics Service, Division of Medical Oncology, National Cancer Centre Singapore, Singapore, Singapore; ^2^Lee Kong Chian School of Medicine, Nanyang Technological University, Singapore, Singapore; ^3^Oncology Academic Clinical Program, Duke-NUS Medical School, Singapore, Singapore

**Keywords:** genodermatoses, cancer genetics, Cowden syndrome, PTEN, cancer predisposition

## Abstract

Cowden syndrome (CS) is an autosomal dominant condition caused by mutations in the phosphatase and tensin homolog (*PTEN*) gene, and is characterized by multiple hamartomas and a predisposition to malignant tumors. Characteristic skin lesions include trichilemmomas, acral keratosis, mucocutaneous neuromas, oral papillomas, and penile macules, and are often the first clues to the underlying diagnosis. Here, we discuss the mucocutaneous manifestations of CS, differential diagnoses of genetic causes of each cutaneous finding, genetic analyses for patients with skin manifestations, management of patients with CS, and potential new targeted therapies for CS.

## Introduction

Cowden syndrome (CS) is a genodermatosis caused by mutations in the phosphatase and tensin homolog (*PTEN*) gene, with an autosomal dominant inheritance pattern. CS is part of a spectrum of disorders caused by germline mutations in the *PTEN* gene, collective named PTEN hamartoma tumor syndrome (PHTS), and includes Bannayan-Riley-Ruvalcaba syndrome (BRRS), Proteus and Proteus-like syndrome (1). The estimated prevalence of CS is ~1 in 200,000 live births, however this is likely an underestimation as the phenotypic variability makes diagnosis challenging (1–3). CS is characterized by multiple hamartomas, and an increased risk of breast, thyroid, endometrial, renal and colorectal carcinomas (2). Characteristic mucocutaneous manifestations of CS include trichilemmomas, acral keratoses, mucocutaneous neuromas, oral papillomas and macular pigmentation of the glans penis (4). As patients with CS can first present with cutaneous lesions with a wide range of differentials, it is important for dermatologists to be aware of the features of CS in order to make the diagnosis. Here, we discuss the mucocutaneous manifestations of CS, differential diagnoses of genetic causes of each cutaneous finding, genetic analyses for patients with skin manifestations, management of patients with CS, and potential new targeted therapies for CS.

## Mucocutaneous Manifestations of Cowden Syndrome

Over 90% of individuals with CS develop some clinical manifestation of the disorder by the late 20s (3, 5, 6); 99% of affected individuals develop mucocutaneous stigmata by the third decade (5).

### Trichilemmomas

Trichilemmomas are benign hamartomatous lesions of the hair follicle outer root sheath (7). Early studies done prior to the establishment of International Cowden Consortium (ICC) criteria reported that all patients with multiple trichilemmomas had CS, and all patients with CS had multiple trichilemmomas (7, 8), however this is likely to be an overestimate due to the original focus on dermatologic manifestations in the diagnostic workup for CS. More recent studies have reported a prevalence of trichilemommas of 6–38% in patients with confirmed *PTEN* mutations (9–12). Clinically, trichilemmomas are well-defined, skin-colored papules 1–5 mm in diameter and located primarily on the face and neck (13). As solitary trichilemmomas can be found in patients without CS (14), the presence of multiple trichilemmomas and confirmation of diagnosis by skin biopsy is needed in order to fulfill criteria for CS (4, 5). Key histological findings are folliculocentric lobular proliferation of polygonal, clear, PAS-positive isthmic cells with nuclear palisading of the peripheral cells on a thickened hyaline eosinophilic basement membrane (14).

In addition to CS, multiple facial papules are also a feature of other inherited conditions. Differential diagnoses for genetic causes of multiple facial papules are summarized in Table 1. Some of these papules have characteristic dermoscopic findings, which can be a helpful aid for clinical diagnosis. For example, trichilemmomas have a radiated red iris-like structure on dermoscopy (15). Other genetic causes of multiple facial papules include Birt-Hogg-Dube syndrome (19–22), multiple familial trichoepitheloma (16–18), Brooke-Spiegler syndrome (16–18), tuberous sclerosis complex (TSC) (23, 24), and Muir-Torre syndrome (25, 26).

**Table 1:** Differential diagnoses for genetic causes of multiple facial papules.

**Skin phenotype**	**Histology**	**Clinical features**	**Dermoscopy**	**Syndrome**	**Gene**	**References**
Trichilemmoma	Folliculocentric lobular proliferation of polygonal, clear, PAS-positive isthmic cells with nuclear palisading of the peripheral cells on a thickened hyaline eosinophilic basement membrane	Skin-colored papules usually 1–5 mm in diameter on face and neck	Radiated red iris-like structure	Cowden syndrome	*PTEN*	(13–15)
Trichoepithelioma	Dermal tumor composed of branched nests of basaloid cells, with keratin cysts and dense collagenous stroma	Multiple firm, dome-shaped papules. Symmetrical distribution on face.	Focused arborizing vessels, pearly white background, milia-like cysts and rosettes	Multiple familial trichoepithelioma, Brooke-Spiegler syndrome	Cylindromatosis tumor suppressor (*CYLD*)	(16–18)
Trichodiscoma	Round to elliptical well-demarcated proliferation of a thick fibrous and vascular stroma in the reticular dermis with a hair follicle at the periphery	2–4 mm smooth, dome-shaped skin/yellow-white papules on face and upper trunk	Nil specific	Birt-Hogg-Dube syndrome	Folliculin (*FLCN*)	(19–21)
Fibrofolliculoma	Round/oval proliferation of spindled cells within a fibromyxoid stroma encasing an epithelial (hair follicle) component, forming elongated retiform extensions within the contiguous dermis.	2–4 mm smooth, dome-shaped skin/yellow-white papules on face and upper trunk	Well-demarcated area of pallor with central small brown spot	Birt-Hogg-Dube syndrome	Folliculin (*FLCN*)	(19, 20, 22)
Angiofibroma	Dermal proliferation of blood vessels, with concentric perivascular fibrosis with stellate stromal cells	Pink-red papules with smooth surface on cheeks and nose, sparing upper lip	Multiple yellowish-white dots distributed over a pinkish-gray background, some with crypts over the surface	Tuberous sclerosis complex (TSC)	*TSC1* or *TSC2*	(23, 24)
Sebaceous adenoma	Sebaceous lobules containing basaloid cells at the periphery and mature sebocytes at the center	Pink or yellow papules with or without a central crater over the face and torso	Lesions with a central crater: elongated crown vessels that surrounded the central crater, which has opaque structureless white-yellow areas Lesions without a central crater: branching, blurred arborizing vessels over a whitish background and yellow globules	Muir-Torre syndrome	*MLH-1 MSH-2*	(25, 26)

### Acral Keratoses

Acral keratoses are located primarily on the dorsum of the hands and feet, and are wart-like, skin colored flat-topped papules 1–4 mm in diameter (27). Palmoplantar keratoses are translucent hard papules with or without a central pit (27). Earlier studies performed before establishment of ICC diagnostic criteria for CS reported a prevalence of acral keratoses of 63–73% in CS patients (27, 28). More recent studies report a prevalence of acral keratoses of 10.2–82% in CS patients with confirmed PTEN mutations (9, 11, 12). Few case reports observed keratosis appearing on non-acral sites such as the face and trunk, but no confirmatory skin biopsies were mentioned (27, 28). Further studies are needed to determine if non-acral keratoses are a common feature in CS.

Histologically, both acral keratoses and palmoplantar keratoses show non-specific orthokeratosis, hypergranulosis, and acanthosis (8). Differential diagnoses of genetic causes of acral keratoses and palmoplantar pits include Buschke-Fischer-Brauer keratoderma (29), Darier's disease (30–32), Cole disease (33), and nevoid basal cell carcinoma (Gorlin) syndrome (34–36) (Table 2).

**Table 2:** Differential diagnoses for genetic causes of acral keratosis, palmoplantar pits, oral papules, mucocutaneous nerve sheath tumors, and penile macular pigmentation.

**Syndrome**	**Histology of selected features**	**Cutaneous features**	**Gene**	**References**
**Differential diagnoses for genetic causes of acral keratoses and palmoplantar pits**				
Cowden syndrome	Acral keratosis and palmoplantar pits: Orthokeratosis, hypergranulosis, acanthosis	Trichilemmomas, acral keratosis, palmoplantar pits, oral papillomas, mucocutaneous neuromas, lipomas, penile pigmentation	*PTEN*	(27, 37)
Buschke-Fischer-Brauer keratoderma	Papules: Orthokeratotic hyperkeratosis, with central depression of underlying malpighian layer, hypergranulosis	Yellow-brown papules with depressed pits on palms and soles	*AAGAB*	(29)
Darier's disease	Acrokeratosis verruciformis: Hyperkeratosis, hypergranulosis, acanthosis, slight papillomatosis and mild perivascular lymphocytic infiltration	Acrokeratosis verruciformis, palmoplantar pits, crusted hyperkeratotic papules over seborrheic areas, mucosal cobblestone, V shaped nicking of nails, oral mucosa papules and cobblestoning over palate, buccal mucosa, tongue	*ATP2A2*	(30–32)
Cole disease	Punctate keratoderma: Hyperorthokeratosis, hypergranulosis, and acanthosis	Punctate keratoderma, hypopigmented macules over arms and legs	*ENPP1*	(33)
Nevoid basal cell carcinoma (Gorlin) syndrome	Palmoplantar pits: Circumscribed zone of hypokeratosis, variable hypogranulosis and parakeratosis, and basal cell hyperplasia with crowding and palisading of basal keratinocytes	Palmoplantar pits, multiple basal cell carcinomas	*PTCH1, PTCH2, SUFU*	(34–36)
**Differential diagnoses for genetic causes of oral papules**				
Cowden syndrome	Oral papilloma: Fibrovascular core covered with benign epithelium which may have regions of hyperplasia	Trichilemmomas, acral keratosis, palmoplantar pits, oral papillomas, mucocutaneous neuromas, lipomas, penile pigmentation	*PTEN*	(8)
Darier's disease	Oral mucosa papule: Suprabasal clefts with focal hyperkeratosis, acantholytic dyskeratosis, corps ronds, grains with perivascular lymphocytic infiltrate	Acrokeratosis verruciformis, palmoplantar pits, crusted hyperkeratotic papules over seborrheic areas, V shaped nicking of nails, oral mucosa papules and cobblestoning over palate, buccal mucosa, tongue	*ATP2A2*	(38)
Tuberous sclerosis complex	Gingival fibroma: Benign squamous epithelium with vascularized stroma	Gingival fibroma, angiofibroma, periungual fibroma, hypomelanotic macule, shagreen patch	*TSC1, TSC2*	(23, 39)
Birt-Hogg-Dube syndrome	Oral papules: Acanthotic epidermis and dense collagenous stroma with few fibroblasts.	Oral papules over lip/buccal/gingival mucosae, fibrofolliculoma, trichodiscoma, acrochordon	*FLCN*	(21)
White sponge nevus	Epithelial thickening, parakeratosis, extensive vacuolization of the suprabasal keratinocytes and compact aggregates of keratin intermediate filaments in the upper spinous layers	Bilateral white, soft thick plaques of the oral mucosa	*KRT13*	(40)
**Differential diagnosis for genetic causes of mucocutaneous nerve sheath tumors**				
Cowden syndrome	Mucocutaneous neuroma: Well-demarcated hypertrophic nerve bundles associated with abundant mucin and surrounded by a distinct perineural sheath	Trichilemmomas, acral keratosis, palmoplantar pits, oral papillomas, mucocutaneous neuromas, lipomas, penile pigmentation	*PTEN*	(41)
Multiple endocrine neoplasia type 2B (MEN2B)	Mucosal neuromas: Hypertrophy of nerves in the dermis, with fascicles of Schwann cells arranged in interlacing patterns	Mucosal neuromas over lips, tongue and buccal mucosa	*RET*	(42, 43)
Neurofibromatosis type 1 (NF1)	Plexiform neurofibroma: Enlarged fascicles within an abundant collagenous matrix Dermal neurofibroma: Non-encapsulated, loosely textured tumor centered on the dermis	Mucocutaneous neurofibromas, café-au-lait macules, skinfold freckling	*NF1*	(44–46)
**Differential diagnoses for genetic causes of penile macular pigmentation**				
Cowden syndrome	Penile pigmentation: hyperplasia of the epidermis with increased pigment in the basal layer and slight increase in the number of melanocytes	Trichilemmomas, acral keratosis, palmoplantar pits, oral papillomas, mucocutaneous neuromas, lipomas, penile pigmentation	*PTEN*	(37, 47)
Carney syndrome	Epithelioid blue nevi: heavily pigmented, poorly circumscribed, dermal lesions that displayed two types of melanocytes: one intensely pigmented, globular, and fusiform; the other lightly pigmented, polygonal, and spindle.	Spotty skin pigmentation (lentigines and epithelioid blue nevi)	*PRKAR1A*	(48, 49)
LEOPARD syndrome	Lentigines: Hyperpigmentation of the basal membrane, increased numbers of melanocytes, slight acanthosis, diffuse lymphohistiocytic infiltrate	Multiple lentigines	*PTPN11*	(50, 51)
Peutz-Jeghers syndrome	Macules: Hyperpigmentation of the basal cell layer, melanocytic hyperplasia, scattered melanocytes and melanophages in the underlying dermis	Mucocutaneous pigmented macules	*STK11*	(52, 53)

### Oral Papillomas

Oral papillomas are 1–3 mm diameter papules which may coalesce, giving the involved surface a cobble-stone pattern (27). Lesions are seen on the lips, buccal mucosae and gingivae; when the dorsum of the tongue is involved, it may take on a scrotal appearance (27). Papillomas have also been reported over the pharynx and larynx, which may require laryngoscopy for identification (54). Earlier studies performed before establishment of ICC diagnostic criteria for CS reported a prevalence of oral papillomas of 83–86% in CS patients (27, 28). More recent studies report a prevalence of oral papillomas of 15.2–85% in CS patients with confirmed PTEN mutations (9, 12). Histology of oral papillomas show a fibrovascular core covered with benign epithelium which may have regions of hyperplasia (8, 54). Differential diagnoses of genetic causes of oral papules include Darier's disease (38), tuberous sclerosis complex (23, 39), Birt-Hogg-Dube syndrome (21), and white sponge nevus (40) (Table 2).

### Mucocutaneous Neuromas

Mucocutaneous neuromas are hamartomas of the peripheral nerve sheath, and present clinically as painful, dome-shaped, translucent pink to skin-colored papules (41). In CS, mucocutaneous neuromas have been reported on the face, hands, trunk, and shin of patients (41, 55). Histology shows non-encapsulated neuromas, with well-demarcated hypertrophic nerve bundles associated with abundant mucin and surrounded by a distinct perineural sheath (41).

Earlier studies performed before establishment of ICC diagnostic criteria for CS reported a prevalence of mucocutaneous neuromas of 5–10.9% in CS patients (27, 28). More recent studies of CS patients with confirmed *PTEN* mutations did not report the incidence of mucocutaneous neuromas (9–12), possibly because the studies were done prior to the addition of mucocutaneous neuromas to CS diagnostic criteria in 2013 (4). In addition, neuromas may be in advertently unreported due to clinical misclassification in the absence of a biopsy (4, 41).

Differential diagnoses for genetic causes of multiple mucocutaneous nerve sheath tumors include multiple endocrine neoplasia type 2B (MEN2B) and neurofibromatosis type 1 (NF1) (Table 2). MEN2B is caused by mutations in the *RET* gene, and is associated with medullary thyroid carcinoma, pheochromocytoma, marfanoid habitus, and multiple mucosal neuromas involving the face (42, 43), as compared to CS, which presents with multiple neuromas affecting acral sites (41). NF1 is caused by mutations in the *Neurofibromin* gene, and is characterized by pigmentary lesions, peripheral nerve tumors (neurofibromas and malignant peripheral nerve sheath tumors), skeletal abnormalities and brain tumors (44). Neurofibromas can be classified into dermal or plexiform neurofibromas (45, 46). A study of 103 patients with NF1 found oral tumors (plexiform neurofibromas or discrete oral neurofibromas), overgrowth of gingival soft tissue and enlarged papillae of the tongue in 74% of NF1 patients (46), which can mimic the oral papillomas seen in CS.

### Penile Pigmentation

Macular pigmentation of the glans penis is a major criterion for CS diagnosis in male patients. The prevalence of pigmented genital macules in the general population is about 15% in males and females (56).

In CS patients with confirmed *PTEN* mutations, the prevalence of macular pigmentation of the glans penis was reported at 19–54.3% (9–12, 57). Histology of penile macules from a patient with BRRS showed hyperplasia of the epidermis with increased pigment in the basal layer and slight increase in the number of melanocytes (47). In a case series of children with PTHS, genital lentiginosis was found in all patients with genital evaluation as early as age 2.5 years (58). A study of patients with PTEN mutations from 1 to 26 years found that penile macular pigmentation was present in 41% of males in the cohort, with earliest report of pigmentation occurring at 15 months of age (57).

Differential diagnoses for genetic causes of penile macular pigmentation include Carney syndrome, LEOPARD syndrome, and Peutz-Jeghers syndrome (Table 2). Carney syndrome is caused by mutations in *PRKAR1A*, presents with spotty skin pigmentation (lentigines and epithelioid blue nevi), endocrine tumors and schwannomas, and can be differentiated from CS by the presence of cardiac myxomas (48, 49). LEOPARD syndrome is named based on the acronym of its manifestations- lentigines, electrocardiographic conduction abnormalities, ocular hypertelorism, pulmonic stenosis, abnormal genitalia, retardation of growth, sensorineural deafness, and is caused by mutations in *PTPN11* (50, 51). Peutz-Jeghers syndrome is characterized by gastrointestinal hamartomatous polyps and mucocutaneous pigmentated macules, which are most often found on the lips, buccal mucosa and digits, but can also be found on genitalia (52, 53).

### Others

Cutaneous features that are associated with CS but not part of major criteria include lipomas and vascular anomalies.

#### Lipomas

The incidence of single lipomas in the general population is 1% (59). In contrast, the incidence of lipomas in CS is 31–39.1% based on early studies without *PTEN* analysis (27, 28), and 34.6–56.7% in patients with CS and *PTEN* mutations (9–11). In order to fulfill minor criteria for CS, a patient has to have at least 3 lipomas (4).

#### Vascular Anomalies

Vascular anomalies have been reported in soft tissue, skin, bone, viscera, and the central nervous system (60) of patients with PTEN mutations. Vascular anomalies in the skin present as hemangiomas, and have a prevalence of 21.7% in early studies (27). Prevalence of arteriovenous malformations in CS patients with *PTEN* mutations in a single study was 6.4–11.4% (61), but prevalence of hemangiomas specifically is not known.

## Criteria for Carrying Out Molecular Analysis

The ICC operational diagnostic criteria for CS was developed in 1996 (62), and has since undergone revisions (1, 6) and incorporation into the National Comprehensive Cancer Network (NCCN) guidelines. Under NCCN 2020 guidelines, patients meeting one of six testing criteria should undergo *PTEN* genetic testing (63). These testing criteria are: (1) Family history of known *PTEN* pathogenic/likely pathogenic variant; (2) Personal history of Bannayan-Riley-Ruvalcaba syndrome; (3) Individual meeting clinical diagnostic criteria for CS [described by Pilarski et al. (4)]; (4) Individual not meeting clinical diagnostic criteria for CS but has a personal history of adult Lhermitte-Duclos disease or autism spectrum disorder and macrocephaly or two or more biopsy-proven trichilemmomas or one of several possible combinations of major and minor criteria; (5) At-risk individual with a relative with a clinical diagnosis of CS/PHTS or BRRS for whom testing has not been performed; (6) *PTEN* pathogenic/likely pathogenic variant detected by tumor profiling on any tumor type in the absence of germline analysis.

Given the multisystem involvement of CS and evolving clinical spectrum, use of the current NCCN criteria may be challenging outside a specialist community. A clinical scoring system based on a prospective study of over 3,000 patients was developed to aid selection of patients for *PTEN* mutation testing (9). The Cleveland Clinic (CC) score is a weighted semi-quantitative score that predicts the individual-level pre-test probability of harboring a germline *PTEN* mutation, based on age-adjusted phenotypic characteristics. Patients with a threshold CC score of 10 (or pre-test probability of 3%) and above are recommended for referral to medical genetics for specialist evaluation. Compared to the NCCN 2010 guidelines, the CC scoring system demonstrated increased sensitivity and specificity for the detection of *PTEN* mutations (9).

Following referral to a medical geneticist, diagnosis of CS is established in a patient by identification of a heterozygous germline pathogenic variant in *PTEN* on molecular genetic testing (5). However, prospective studies estimate that only ~25% of CS patients who meet clinical criteria harbor pathogenic *PTEN* mutations (9, 64, 65). For patients who meet CS clinical diagnostic criteria and have known familial pathogenic variants, genetic testing for familial pathogenic variants is conducted. If these patients are positive for familial pathogenic variants, management and cancer surveillance should be performed as per NCCN guidelines for CS. If these patients are negative for familial pathogenic variants, cancer screening should occur as per NCCN screening guidelines (63). For patients who meet CS clinical diagnostic criteria and do not have known familial pathogenic variants, genetic testing using a multi-gene panel should be conducted. If patients are positive, management and cancer surveillance should be performed as per NCCN guidelines for the appropriate gene. If patients are negative, they should be managed as likely pathogenic variant carriers (63).

Research efforts to elucidate non-*PTEN* etiologies for CS have identified *SDHx, KLLN, AKT1, PIK3CA, SEC23B*, and *WWP1* as candidate predisposition genes for CS (66–70), but these genes have yet to be validated for use in a clinical setting. Identification of susceptibility genes is important because it allows patients to have gene-specific genetic counseling, predictive testing of family members and precise risk assessment.

## Surveillance

As CS is associated with increased risk of breast, endometrial, thyroid, gastrointestinal and renal cell carcinoma, cancer screening is a crucial component of management, and has been described in NCCN guidelines (63). Briefly, women should begin regular breast self-examination at age 25 years, and commence annual mammography starting at age 30–35 or 5–10 years before onset of known breast cancer in the family. Endometrial cancer screening should be decided on an individual basis starting at age 35 years. Annual thyroid ultrasound should start at age 7 years. Colonoscopy should start at age 35 or 5–10 years before earliest colon cancer in the family, and repeated at least every 5 years. Renal ultrasound should be considered every 1–2 years from age 40 years. Although data on melanoma risk in CS is limited, there is reported increased melanoma standardized incidence risk (SIR) of 8.5 in *PTEN* mutation positive CS patients (61), with median age of diagnosis of 40 years, compared with 63 years in the general population (71). Hence, annual dermatology examination of CS patients is recommended.

## Management of Mucocutaneous Lesions

Treatment of cutaneous manifestation of CS is the same as for their sporadic counterparts (5). Trichilemmomas are benign lesions and do not require treatment, although carbon dioxide laser ablation or simple surgical excision have been utilized (72). There are case reports of the use of oral retinoids for treatment of oral papillomas or acral keratosis, but the lesions recurred following cessation of treatment (73, 74). Treatment of severe oral papillomatosis include surgical excision, carbon dioxide laser debulking, or localized radiotherapy (75).

## Discussion

### New Potential Targeted Therapies for Skin Lesions

PTEN functions both in the cytoplasm to regulate signaling pathways, and in the nucleus to maintain chromosomal stability and DNA repair (76). PTEN is a dual-specificity phosphatase that dephosphorylates both protein and lipid substrates. For example, PTEN dephosphorylates focal adhesion kinase (FAK), resulting in inhibition of cell migration (77). PTEN also negatively regulates the PI3K/AKT/mTOR pathway. In the absence of PTEN inhibition, phosphorylation of phosphatidylinositol 4,5-bisphosphate (PIP2) to phosphatidylinositol 3,4,5-triphosphate (PIP3) causes AKT activation, resulting in phosphorylation of proteins such as mTOR that affect cell growth, cell cycle entry and cell survival (78). PTEN dephosphorylates PIP3 to PIP2 (79), hence loss of PTEN lipid phosphatase activity results in constitutive activation of the PI3K/AKT/mTOR pathway.

Inhibition of mTOR is thus a reasonable therapeutic strategy. A pilot study involving 18 CS patients showed that administration of oral mTOR-inhibitor sirolimus 2 mg daily for 56 days was tolerable in terms of side effect profile, and was sufficient to down-regulate mTOR signaling in skin and gastrointestinal tissue (80). Of note, 14 patients demonstrated improvement in skin lesions based on dermatologic examination, although the specific lesion type (trichilemmoma, oral papilloma, etc.) or method of assessing improvement were not mentioned and will require larger patient populations for confirmatory studies (80). One limitation of targeting the PI3K/AKT/mTOR pathway is feedback activation of other signaling pathways, causing drug resistance (81). In theory, this could be overcome by the use of combination drug therapies. For example, rebound increase in AKT activation has been observed in rapamycin-resistance breast cancer cell lines, and this can be overcome with concomitant administration of resveratrol (82).

Given the myriad *PTEN* mutations and resultant diverse disruption in cellular function, a possible approach is to target therapeutics to the specific mutation each patient has. For example, mutation p.Lys289Glu identified in a CS patient resulted in a nuclear import defect of PTEN (83). Nuclear PTEN is involved in DNA double-stranded break repair (76), and loss of PTEN causes increased sensitivity of cells to the inhibition of DNA repair enzyme PARP1 both *in vitro* and in mouse models, resulting in reduced cell growth (84). However, given that *PTEN* germline mutations in CS affect all cells, targeting the PAPR1 inhibitor to tumor cells would be key.

## Conclusion

Mucocutaneous manifestations of CS may appear similar to that of other inherited conditions, hence it is imperative for dermatologists to be aware of the constellation of signs that typify CS. Expedient referrals to cancer geneticists for genetic testing and initiation of cancer screening is paramount. Further studies to identify organ-specific therapeutic strategies for CS are still needed.

## Author Contributions

All authors contributed to the conceptualization, writing, and editing of the manuscript.

## Conflict of Interest

The authors declare that the research was conducted in the absence of any commercial or financial relationships that could be construed as a potential conflict of interest.
